# Are Ischemic Stroke and Alzheimer’s Disease Genetically Consecutive Pathologies?

**DOI:** 10.3390/biomedicines11102727

**Published:** 2023-10-08

**Authors:** Ivan B. Filippenkov, Andrey V. Khrunin, Ivan V. Mozgovoy, Lyudmila V. Dergunova, Svetlana A. Limborska

**Affiliations:** Laboratory of Human Molecular Genetics, National Research Center “Kurchatov Institute”, Kurchatov Sq. 2, 123182 Moscow, Russiakhrunin-img@yandex.ru (A.V.K.); ivmstalker@gmail.com (I.V.M.); dergunova-lv.img@yandex.ru (L.V.D.)

**Keywords:** ischemic stroke, Alzheimer’s disease, consecutive pathologies, functional genomics, transcriptomics, non-coding RNAs

## Abstract

Complex diseases that affect the functioning of the central nervous system pose a major problem for modern society. Among these, ischemic stroke (IS) holds a special place as one of the most common causes of disability and mortality worldwide. Furthermore, Alzheimer’s disease (AD) ranks first among neurodegenerative diseases, drastically reducing brain activity and overall life quality and duration. Recent studies have shown that AD and IS share several common risk and pathogenic factors, such as an overlapping genomic architecture and molecular signature. In this review, we will summarize the genomics and RNA biology studies of IS and AD, discussing the interconnected nature of these pathologies. Additionally, we highlight specific genomic points and RNA molecules that can serve as potential tools in predicting the risks of diseases and developing effective therapies in the future.

## 1. Introduction

Diseases that affect the functions of human higher nervous activity and are accompanied by severe cognitive impairment account for a significant proportion of diseases recorded in human populations. The number of cases of such diseases has been rapidly increasing in recent years, due to socio-economic cataclysms and the general aging of the population [1,2]. Neuropathology is generally considered as one of the main health problems, which determines the high social significance and relevance of research aimed at elucidating the causes of such diseases, as well as developing systems for preventing and correcting disorders of human brain activity. According to the World Health Organization, ischemic stroke (IS) and Alzheimer’s disease (AD) occupy leading positions among brain neuropathologies [3,4,5]. Clinically, IS is a consequence of a permanent or temporary decrease in cerebral blood flow, mostly caused by the occlusion of cerebral arteries by a thrombus or embolus [3,6,7,8]. AD, ranking first among neurodegenerative diseases in the world, is characterized by loss of memory and other cognitive functions, and leads to profound dementia [9,10]. The relationship between these diseases can be discussed from several perspectives: clinical (as comorbidity), pathological (as shared pathological processes) and genetic (as the overlap of genetic markers and transcriptomic changes).

IS is considered a risk factor for AD by, as an example, the American Stroke Association [11]. In a meta-analysis by Zhou et al. [12], pooled data showed that the risk of AD after a stroke was increased by up to 60% (pooled effect size of 1.59). The reverse causation, the occurrence of IS in AD patients, was not found to be significant in this article or in another study [13], but was reported in a more recent meta-analysis by Pinho et al. [14]. Additionally, all the mentioned papers demonstrated that AD is a risk factor for intracerebral hemorrhage (ICH) with a pooled effect size of 1.42–1.41. Furthermore, IS and AD share many of the same risk factors, including hyperlipidemia, hypertension, heart diseases, diabetes and obesity [15,16,17].

The profile of brain neurodegeneration observed after ischemia shares common features with neurodegeneration in AD. Firstly, it is characterized by the accumulation of beta-amyloid peptides in the extracellular space of the brain. This accumulation occurs due to their influx from the blood and/or impaired clearance from the brain, which is caused by the disruption of the blood–brain barrier (BBB) [18,19,20,21]. Additionally, ischemic brain injury has been shown to lead to dysfunction of the tau protein, which can be initiated by beta-amyloids, neuroinflammation or blood-borne tau infiltrated through the disrupted BBB [22,23,24]. Furthermore, pathogenic consecutiveness comes from the fact that both AD and IS diseases have common secondary pathological processes, such as neuroinflammation and excitotoxicity [25]. As a result, there are some similarities between the pathological phenotypes [26].

At the molecular level, the profiles of neurodegeneration (brain damage) that form during the development of AD are in many aspects similar to those observed after IS [27,28,29,30,31,32,33,34]. Currently, there are data on the role of molecular genetic factors in the development of AD. However, the identified loci only account for a small proportion of the observed phenotypic variations. This highlights the particular importance of using genomic analysis methods to identify the so-called “lost” heredity, i.e., currently unknown, but potentially significant, DNA sequence variants, patterns of genomic architecture and regulatory nodes on a postgenomic level. Taking an integrated approach to studying the functioning of the brain after IS can provide insights on the mechanisms of brain degeneration in AD and vice versa, considering AD and IS as interconnected diseases.

In this review, our aim was to investigate the molecular crosstalk between IS and AD and to illustrate its complexity. To achieve this, we summarized studies on the genomics and transcriptomics of both diseases, paying special attention to role of non-coding RNAs, as these molecules show promise as tools for diagnostics and treatment.

## 2. Materials and Methods

In this review, we included clinical trials published by PubMed until 20 June 2023. The keywords used were “ischemic stroke”, “Alzheimer’s disease”, “genomics of stroke”, “genomics of Alzheimer’s disease”, “ischemic stroke and transcriptomics”, “Alzheimer’s disease and transcriptomics”, “microRNA” “microRNA” and “Alzheimer’s disease”, “microRNA and ischemic stroke”, “microRNA profiling”, “circRNA and Alzheimer’s disease” and “circRNA and ischemic stroke”.

All included studies were selected from peer-reviewed journals. MicroRNA–mRNA or circRNA–microRNA interactions in the corresponding sections and tables were obtained only from the articles containing experimental validation of these interactions, not just prediction by bioinformatic tools.

Articles from non-peer-reviewed journals, retracted studies, without available abstracts or English translations were excluded.

## 3. Results

### 3.1. Alzheimer’s Disease (AD)

A significant challenge in the diagnosis and treatment of AD is that clinical symptoms only become apparent several years after the onset of pathological processes in the brain. By the time a diagnosis is made, there is already progressive degeneration of neurons due to the destruction of their cytoskeleton. Most researchers suggest that AD may be associated with the accumulation of beta-amyloid and the formation of neurofibrillary tangles in the cerebral cortex and subcortical gray matter [21,35,36,37]. Specific cases of the disease have been associated with mutations in the amyloid precursor protein gene, in the presenilin genes and some others [38,39,40,41]. Additionally, the presence of the ε4 allele of the APOE gene has found to be significant. However, only a small percentage of AD cases are hereditary [42,43,44]. The majority of cases (over 90%) are sporadic forms of AD with late onset and an unspecified etiology [10,38].

### 3.2. Ischemic Stroke (IS) and AD

Recent studies have shown that acute IS may be a significant risk factor for the development of sporadic forms of AD (as a common trigger for AD) [45,46,47]. Conversely, an increase in the risk of IS has also been observed in the context of AD development [27,28,29,30,31,32,33,34]. Additionally, it has been reported that AD and IS often occur consecutively [30,32,33,34,48]. The profile of brain neurodegeneration observed after ischemia shares common features with neurodegeneration in AD. Studies have found that cerebral ischemia, in both humans and animals, leads to the accumulation of beta-amyloid peptides in the extracellular space of the brain [18,19,20,21]. Moreover, the tau protein, which is an important marker of AD, may exacerbate brain damage in an animal model of stroke by mediating excitotoxic Ras/ERK signaling [34,49]. Additionally, it has been demonstrated that ischemic brain injury disrupts the metabolism of the tau protein, which enters the brain when the blood–brain barrier is disrupted [30,32,33,34,48]. However, a notable study by Koenig et al. was recently published [50]. African American and non-Hispanic white stroke patients from Saint Louis, MO and controls from an AD research center were studied using MRI, PET and other clinical and cognitive measures [50]. The authors did not find evidence that preclinical AD is a risk factor for stroke or predicts post-stroke dementia, supporting the idea that vascular disease and amyloid pathology are separate disease mechanisms that may each lead to dementia. The authors note some limitations of the representatives of APOE4 genetics in cohorts as well as the limited statistical power due to the small number of participants [50].

### 3.3. Genome-Wide Association Studies of IS

The genetic component is of particular interest as hereditary variations can affect not only the risk of stroke itself, but also determine the potential risk of traditional factors. Therefore, this study focuses on the genes associated with the onset of IS in humans. Similar to AD, it is increasingly clear that the influence of a number of molecular genetics needs to be considered when assessing the development of IS [51,52]. Through candidate genes analysis and genome-wide studies of single nucleotide polymorphism (Genome-Wide Association Studies, GWAS), a number of genes associated with the risk of stroke have been identified [53,54]. The first GWAS results demonstrated individual associations between specific polymorphic variants with specific pathophysiological subtypes of strokes. For instance, polymorphisms in the *PITX2* and *ZFHX3* genes were associated with cardioembolic stroke, while markers in the 9p21 locus and the *HDAC9* gene were associated with atherothrombotic stroke [55,56,57]. Subsequent studies have already identified polymorphisms (genes) associated not only with individual IS subtypes, but also with strokes in general [58,59]. Many of the identified loci also showed associations with other signs that are risk factors for IS (blood pressure, atrial fibrillation, lipid levels, etc.) [53,58].

With an increase in the size of the analyzed groups of patients and controls, the number of IS risk loci identified in studies has also increased. This increase made it possible to discover new mechanisms and pathways involved in the development of stroke. For example, within the largest GIGASTROKE project to date, 89 genomic loci were identified, with 61 of them being described for the first time [60]. However, the clinical effectiveness of these loci remains unclear, as they only account for a small percentage of the cumulative phenotypic variations, ranging from 0.5 to 2% depending on the stroke subtype and ethnicity of the analyzed population [58]. Losses are implied by the limitations of traditional statistical methods based on conducting a huge number of single-marker tests that satisfy a certain level of significance, and their inability to take into account the complexity of relationships between genome elements, both due to the presence of linkage disequilibrium and possible interactions between genes (epistasis) [61]. Artificial intelligence methods are expected to help overcome these limitations and improve the results of GWAS. By using more sophisticated data processing algorithms, these methods offer an alternative to classical statistics used in GWAS studies [62]. Using neural networks with the “autoencoder” architecture, Chinese scientists predicted 10 new stroke candidate genes using the protein–protein interactions (PPI) network and a list of stroke-associated genes [63]. Functional analysis of the predicted genes revealed their relevance to stroke symptoms.

### 3.4. GWAS of AD

Similar GWAS studies have also been conducted in groups of patients with AD. To date, approximately 95 loci have been identified, with polymorphisms associated with the risk of developing AD [64]. Suggested in early GWAS, almost all pathways that were critical to AD development, including the Aβ pathway (*APP*, *PSEN1,* and *PSEN2*), inflammatory response (*CR1*, *CD33*, *MS4A*, *ABCA7*, *EPHA1*, *TREM2* and *CLU*), lipid metabolism (*APOE*, *SORL1*, *ABCA7* and *CLU*) and endocytosis/vesicle transport (*BIN1*, *CD2AP*, *PICALM*, *EPHA1* and *SORL1*) [31,65,66,67,68,69,70,71,72,73]. Further progress in AD research was achieved with the use of an alternative GWAS approach that includes not only subjects with a defined disease (cases), but also subjects without a disease (controls) and their relatives (proxy cases and proxy controls) [73,74,75,76]. Data from these larger GWAS, along with functional genomics, have highlighted the significant role of microglia in AD. Recently, variability in microglia-related genes has also been determined to be a major contributor to AD heritability (accounting for 69–84% of total heritability), further emphasizing its importance in AD development [77].

### 3.5. Crosstalk between GWAS of IS and AD 

Recent studies have indicated that there are overlapping parameters in the genetic architecture between AD and strokes [28,29,78]. Taylor et al.’s research suggested a common genetic predisposition to AD and small vessel stroke, identifying four associated pathways, including cholesterol transport and immune response [29]. Another study, based on the analysis of two large GWAS statistics for AD (17,008 AD cases and 37,154 controls) and IS (10,307 stroke cases and 19,326 controls) [78], identified 16 pleiotropic genes that were significantly associated with both diseases. Many of them (*EPHA1*, *MS4A4A*, *UBE2L3* and *TREM2*) were related to the functioning of the immune system. These findings emphasize the crucial role of the immune response in the pathogenesis of AD and IS. Notably, two established AD susceptibility genes, *MS4A4A* and *TREM2*, were found to be significantly altered in ischemic spleen and brain, respectively [78].

### 3.6. Transcriptomics of IS

With the rapid development of genome-wide analysis methods and multi-omics technologies, studying of the post-genomic levels of regulation of gene functioning has become possible. Transcriptome-level analysis is of significant importance, as it allows for the comprehensive assessment of genomic loci that are involved in the formation of disease-associated phenotypic parameters.

In 2006, Ford et al. conducted microarray profiling of two (permanent and transient) middle cerebral artery occlusion (MCAO) rat models [79]. This study identified genes associated with each of the two IS models. Genes unique to transient MCAO were mainly involved in the induction of inflammatory and oxidative stress, while permanent MCAO led to the expression of genes more associated with metabolic activity and cell signaling [79]. Subsequently, the transient MCAO (tMCAO) model was used to reveal the differential expression of multiple genes not only in the focal areas, but also in adjacent brain regions using RNA-Seq and NanoString nCounter technologies [79,80,81,82,83,84,85,86]. Recently, transcriptome and immunohistochemical approaches have shown that acute IS triggers a cellular senescence-associated secretory phenotype [87].

Interestingly, the gene expression profile after a stroke, as shown, is subject to temporal control. A study on mice revealed changes in the mRNA level of cytokines. TNF-a, IL-1b, IL-10 and TGF-b1 genes increase their expression level in the first hours after tMCAO, but return to the healthy control level a day later. At the same time, the mRNA of the HSP-70 heat shock protein gene remains significantly elevated and 24 h after occlusion [88]. A microarray of RNA from rat blood samples was conducted at 0, 1, 2, 3, 6 and 24 h after tMCAO. Multiple stereotyped and time-dependent profiles of gene expression were identified within 24 h. As noted in the study, temporally overlapping profiles have the potential to provide a biological “stroke clock” to stroke prevention [89]. Additionally, mRNA expression of genes for inflammatory Iba-1, CD68, CD16 and CD86 proteins was increased in the ipsilateral olfactory bulb compared to the contralateral side 3 days after tMCAO in rats [90]. Recently, gene expression changes in cells during neuroinflammation were identified 24 h after tMCAO using single cell sequencing (scRNA-Seq). The authors identified 17 principal brain clusters with cell-type specific gene expression patterns as well as specific cell subpopulations and their functions in various pathways [91]. In addition, the next study discovered at least six microglial subsets in the stroke-aged brain (19–20 months old mice), including a potentially stroke-specific subtype using scRNA-Seq and transcranial tMCAO [92].

### 3.7. Transcriptomics of AD

As part of the study of the molecular genetic mechanisms of AD, studies of gene expression were carried out under the conditions of the corresponding models of transgenic mice. Different authors have shown a predominant increase in the expression of immune system genes with the development of amyloid plaques in the hippocampus and cerebral cortex [93,94,95,96,97]. Using microarrays under conditions of four lines of “amyloid” transgenic mice (mutant human APP gene, *PSEN1* or *APP*/*PSEN1*) and the transgenic mice “TAU” (mutant human MAPT gene), researchers have demonstrated that the expression of immune system genes correlated with the presence of plaques. Conversely, genes associated with synaptic signaling showed a negative correlation with neurofibrillary tangles [98]. Transcriptome analysis of the APPswe/PS1 L166P and Thy-TAU22 models in mice showed that the *APOE*, *CLU*, *INPP5D*, *CD33*, *PLCG2*, *SPI1* and *FCER1G* genes, which are among the AD risk genes, significantly upregulate expression when exposed to beta-amyloid. Furthermore, sequencing of single microglia cells confirmed noticeable transcriptional changes in microglia, including an increase in the proportion of activated microglia, caused by the pathology of amyloid beta, not the tau protein [99]. This study indicates that the risk of sporadic AD is associated with genes expressed in microglia, which respond to beta-amyloid deposition. At the same time, astrocytes, neurons and oligodendrocytes also exhibit different genome responses to amyloid plaques [100]. A recent large-scale analysis of spatial transcriptomics (ST) and in situ sequencing (ISS) methods on the brain samples of mice and humans, revealed a coordinated genome response in AD conditions. Two main groups of genes were identified. The first group consisted of genes related to myelination and the functioning of oligodendrocytes (*Plp1*, *Mbp*, *Mobp*, *Cldn11*, *Mal*, *Apod*, *Cnp*, *Trf*, *Fth1*, etc.), which showed early changes in activity. The second group included genes associated with complement systems, lysosomes, inflammation and the response to oxidative stress. They exhibit activity in a noticeably later phase of AD [101]. Transcriptomic data on AD are currently enriched to a large extent using new single-cell sequencing methods (single-cell/single-nucleus RNA-Seq) [102,103].This data have already allowed researchers to propose novel potential treatment strategies, such as treatments based on ferroptosis inhibition [104].

### 3.8. Crosstalk between Transcriptomic Data of IS and AD

Researchers have attempted to compare the transcriptomic profiles of IS and AD. There are at least two papers where whole-transcriptome comparative data are provided. Liu et al. conducted a study on a large subset of peripheral blood samples of patients with IS and AD [105]. They identified 74 genes that are differentially expressed in both diseases, including *APOE*, *SOD1* and *RPS3*. Many of the genes were found to be related to the immune system. The authors call it the “crucial mechanism behind the correlation between AD and IS”. Another group performed a comparison between tMCAO and 5xFAD mice [106]. In total, 401 genes were identified, including recently reported common genes such as *TREM2* [107]. The functional annotations of these genes were also related to the immune system.

### 3.9. MicroRNAs in IS and AD

To date, it has been demonstrated that not only coding mRNAs, but also various types of non-coding RNAs (ncRNAs), molecules with significant regulatory potential, are involved in the response to pathological effects [108,109,110,111,112]. One of the extensively studied types of ncRNAs is microRNAs (miRNAs). MiRNAs are RNA molecules that are 18–24 nucleotides in length, often transcribed from intron regions of genes and capable of degrading the target mRNA or limiting its translation. Bioinformatically, more than 60% of mammalian mRNAs have conserved target sites for at least one miRNA [113].

To date, there is a significant amount of data available on the differential expression of miRNAs in pathological conditions. For IS, miRNA profiles are provided in the blood of patients [114,115] and in brain tissues [116]. MiRNA profiles were also obtained for rats after tMCAO in the blood and in various tissues, including different areas of the brain [117]. Profiles are also available for oxygen glucose deprivation/re-oxygenation (ODG/R)-induced cell cultures, including astrocytes [118]. For AD, miRNA profiles are also revealed in serum and cerebrospinal fluid (CSF) [119] and the hippocampus [120] of AD patients. For various patient tissues, expression data are provided by Takousis et al. [121] and Yoon et al. [122]. MiRNA expression profiles have also been obtained for in vivo models—such as for the cortex of APP/PS1 mice [123]. Several reviews can provide a systematic understanding of the role of miRNAs in IS [124] and AD [125,126] and can serve for obtaining transcriptomic data.

Table 1 highlights crosstalk in miRNA-mediated regulation between IS and AD. The inclusion of miRNA in the table were based on the availability of the information about expression changes in both diseases (or in their models), the presence of experimentally confirmed mRNA-targets encoding pathologically significant proteins and confirmed interactions with differentially expressed circRNAs in the respective diseases.

It is important to note that some miRNAs are associated with IS and AD due to the commonality of the pathological processes underlying both diseases (apoptosis, neuroinflammation, oxidative stress). For instance, miR-125 and miR-211 are associated with apoptosis, and miR-125 regulates inflammation in both diseases (Table 1). Additionally, certain miRNAs are involved in disease-specific pathologic processes. An example is miR-23a-3p. Jiang et al. showed that this miRNA interacts with the mRNA of GSK-3β, a kinase important for tau protein phosphorylation in AD conditions; whereas, in IS model conditions, the pro-apoptotic role of miR-23a-3p was shown. Another unique function of this miRNA in IS is the prevention of oxidative stress after reperfusion [155]. For miR-103, its role in the regulation of apoptosis has been shown in animal models of both diseases; however, miR-103 interaction with vascular endothelial growth factor (VEGF) mRNA, as well as preventing excitotoxicity by targeting the transcript of the Na^+^/Ca^2+^ exchanger gene (NCX1), has been shown only in IS. Overall, five of the nine described miRNAs have disease-specific functions reported to date, and we can expect new evidence about these unique contributions of miRNAs in IS and AD.

Third, six of nine miRNAs change their expressions in opposite ways. Possible explanations are limited by the fact that all models, in which these changes are observed, are not completely comparable. One model may show the compensation state of some pathological process, while another will show the decompensation state. As an example, even in one animal model we have significant spatial [117] and temporal [156] transcriptomic changes.

### 3.10. Circular RNAs in IS and AD

MiRNAs regulate the expression of target miRNA, but they themselves are also regulated by various types of non-coding RNAs (ncRNAs). A new type of covalently closed molecules of non-coding RNAs (circular RNAs, circRNAs) has attracted special attention due to their properties, which are fundamentally different from those of other types of RNA. First, circRNAs have increased metabolic stability, as the lack of free 5′ and 3′ ends [157,158]. Second, circRNAs often originate from protein-coding genes along with mRNA, but do not code proteins and may be subject to a different regulation of expression than mRNA [159,160]. Third, circRNAs are highly abundant in brain tissue cells [161,162,163,164,165]. Fourth, it has been shown that circRNAs are actively expressed under various disease conditions [156,166,167,168,169,170]. Additionally, circRNAs are highly homologous in humans and rodents (common model animals) [171]. Therefore, circRNAs are an important focus of translational research. The functional significance of circRNAs continues to be actively studied. The ability of circRNAs to interact with miRNAs, neutralize their activity and thereby prevent the miRNA-mediated repression of protein-coding transcripts has been most proven [165,172].

CircRNA expression and their functions in different pathological conditions are actively studied. On one hand, there is strong evidence of their importance and therapeutic perspectives [173]. On the other hand, there is lack of sufficient data, lack of established approaches and even a lack of consensual nomenclature. The paper by Vromman et al. [174] can provide a good presentation of these problems, and all of them can be perceived as important and actual research goals.

For IS, circRNA expression data are available for tMCAO rats [156] and the blood of IS patients [175]. There are also some up-to-date reviews that can lead to a deeper understanding of circRNA’s contribution to IS [176,177]. Table 2 illustrates some circRNAs associated with IS. All of them have some validated targets and functions in IS conditions.

For example, ciRS-7 encoded by the CDR1 gene is involved in the miR-7/α-Syn (SNCA) axis; the overexpression of ciRS-7 suppresses α-Syn protein induction and promotes motor function recovery, decreases infarct size and curtails the markers of apoptosis, autophagy and inflammation in the post-stroke brain [178]. Additionally, circTLK1 is involved into miR-335/TIPARP axis. The knockdown of circTLK1 decreased infarct volumes, attenuated neuronal injury and improved neurological deficits in tMCAO mice [150]. A similar effect can be achieved with the knockdown of circHECTD1; it is involved in the miR-335-3p/TIPARP axis. Gene TIPARP, regulated by this axis, is involved in astrocyte activation [139].

For AD, there are experimental data available for the frontal cortex [179]. Computational analysis has revealed circRNAs, which may be associated with AD pathology [180]. There are also reviews about circRNAs in AD [181]. Table 3 illustrates some circRNAs associated with AD.

In AD conditions, there is a reported age-dependent loss of circHDAC9. This circRNA is known to be part of circHDAC9-miR-138-APP/PS, and the decreased expression of its target protein activated Aβ production.

Several differentially expressed circRNAs reported to be differentially expressed in IS and AD are presented in Table 2 and Table 3, respectively. These data are presented separately because only one circRNA is reported to be differentially expressed in both conditions. However, each of the shown circRNA has a validated interaction with miRNA involved in both IS and AD (and described in Table 1). In both tables, the majority of circRNAs have lowered expression compared to one in a healthy state. This aligns with the experimental results obtained in our lab: whole-genome RNA sequencing revealed 377 downregulated circRNAs and only 18 upregulated circRNAs in the rat brain under tMCAO conditions [156]. An expression of target miRNA may be expected to change in the opposite direction from its circRNA “sponge”. It can be observed that this assumption is often (but not always) consistent with the data provided in Table 2 and Table 3.

The only circRNA reported to be differentially expressed in both IS and AD is ciRS-7, one of the first and most described molecules of this type [184]. However, due to the lack of data, especially on circRNAs in AD, it is possible to believe that more similarities are yet to be discovered.

## 4. Discussion

The relevance of studying the mechanisms underlying the commonality of brain diseases is driven by the current demographic transition, advances in medicine and the increase in the average life expectancy of the population. As a result, the older population is experiencing a wider range of diseases that often overlap, modifying disease trajectories. Therefore, investigating the genetic basis of non-random combinations of multifactorial diseases, such as AD and IS, is not only important for understanding the underlying pathogenetic mechanisms, but also for the development of new approaches to assess and prevent risks for both patients and their relatives. To further advance our understanding of the genetic relationship between AD and IS, it is necessary to apply functional genomics methods to study AD risk genes in the context of ischemic brain damage and, conversely, IS risk genes in conditions of AD. Although studies focused on the role of the tau protein have been conducted [27,48,185,186], the available research data are insufficient for a comprehensive understanding of the relationship between the etiology of AD and strokes. Therefore, it is a prospective task to investigate the structure and function of the genes involved in AD and IS, in both humans and in model systems using comparative genomics, transcriptomics, biomedicine, as well as innovative approaches from the fields of physical and computer science.

**Figure 1:**
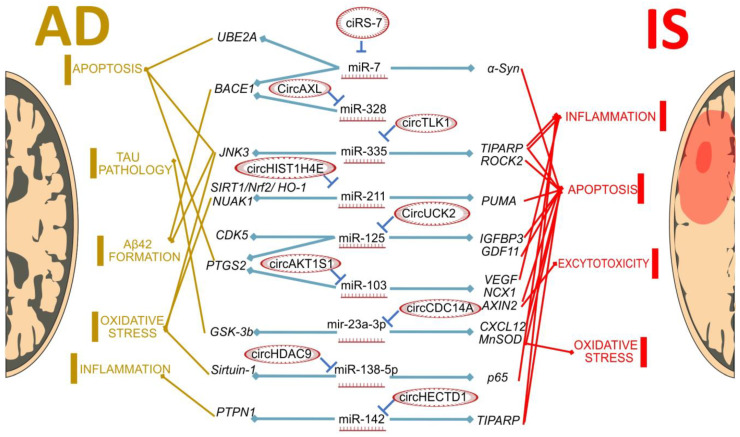
MiRNA–circRNA axes in IS and AD. All miRNAs in the center of the figure are differentially expressed in both IS and AD model conditions. CircRNAs on the left are differentially expressed and associated with AD, and circRNAs on the right—with IS. All circRNA/miRNA/mRNA interactions are experimentally validated.

A modern search for methods to prevent IS and AD should also consider utilizing the properties of new RNA types. The contribution of experimentally validated miRNA-circRNA axes to the pathogenesis of IS and AD is graphically summarized in Figure 1. Although these data are illustrative and focus only on two types of ncRNAs, there are much more relevant types of ncRNAs, such as long non-coding RNAs [187,188]. Nevertheless, it is sufficient for understanding the importance and perspectives of further studying the role of ncRNAs in IS and AD.

It is important to note that the data were obtained from different models. tMCAO rats are considered suitable for preclinical stroke research [189,190], while the APP/PS1 mice model is used for AD research [191]. Although the suitability of IS [192] and AD [193] models is still being debated, we still need to refer to them to obtain most of the transcriptomics data. It is important to consider all limitations when translating these results to humans. Another limitation is the conflict on the direction of expression changes in different models. As shown in Table 1, there are molecules that change expression in opposite ways in animals, cell models and human cells. This controversial fact is discussed further below. We can speculate about the significance of these RNA axes in the corresponding diseases, but more data on circRNA expression and changes in pathology are still needed.

As seen in Figure 1, there are several common pathological processes involved in both IS and AD, such as apoptosis, oxidative stress and neuroinflammation. Genes that are involved in the development of these processes are sometimes regulated by the same miRNAs. For example, the miR-7, miR-335 and miR-125 target genes are involved in apoptosis, while mir-142 regulates genes related to neuroinflammation in both conditions. However, there are differences in the circRNAs that interact with these miRNAs, between IS and AD. This may highlight the differences between these two pathological conditions: as an example, circAXL/mir328/BACE1 regulates Aβ production, and this process is AD-specific. So, circRNA can contribute to the development of disease-specific changes in the transcriptome level. However, we can also expect more similarities in the circRNA profiles yet to be found, as shown in the CirS-7/miR-7 axis, involved in regulation apoptosis in both AD and IS. Both common and unique circRNAs and circRNA–miRNA–mRNA axes may have important implications in the diagnostics and treatment of brain disorders.

The study and application of the circRNAs properties in the context of IS and AD is highly relevant. This is primary due to the diverse and unique properties of circRNAs, which hold promise for enhancing the existing methods for preventing IS and AD as well as managing the consequences of these diseases [194,195]. Moreover, the properties of circRNAs as new important regulators in the nervous system may underlie the phenomenon of the interconnectedness of neuropathologies, in particular IS and AD. This aspect, in our opinion, is particularly promising and warrants further investigation.

It should be noted that, currently, there are very few safe and effective therapies for IS and AD therapy [196,197]. However, many believe that there may be circRNAs with potential therapeutic properties. For example, Yang et al. showed that circSCMH1, when delivered via extracellular vesicles, binds to the MeCP2 protein, leading to the removal of the repression of downstream MeCP2 target genes. There is a significant increase in neuroplasticity and the inhibition of glial reactivity and peripheral immune cell infiltration in rodents and primates after a stroke [198]. Additionally, the delivery of circDYM via extracellular vesicles alleviates depressive-like behavior induced by chronic unpredictable stress in mice [199]. Indeed, the world is still far from a safe and effective technology for circRNA-based therapies, and there is a clear lack of fundamental scientific data. Nevertheless, as the review shows, there are convincing perspectives to overcome these data gaps.

## 5. Conclusions

From the analysis of the literature, it becomes clear that the combination of genomic and transcriptomic approaches is one of the effective ways to study the features of the genesis of complex socially significant diseases, including AD and IS. RNA molecules and their axes can play a regulatory role in genome functioning and pathogenesis in brain cells. circRNAs can serve as new potential regulators in the brain during IS and AD. It is possible that the properties of this class of RNAs may underlie the phenomenon of the IS and AD as interrelated pathologies. Thus, circRNAs can be a promising tool in predicting the risks of disease and creating effective therapies in the future.

## Figures and Tables

**Table 1:** MiRNAs associated with both IS and AD *.

MiRNA	Disease	Differential Expression Data *	Validated Targets	Target Function	References
MiR-7	IS	tMCAO rats ↓	α-Syn	Promotes neuronal death	[127]
	AD	PBMCs of AD patients ↑	BACE1	Required for the generation of all forms of Aβ, including Aβ42	[128,129,130,131]
MiR-125	IS	OGD/R triggered BV2 cells ↓	IGFBP3	Promotes apoptosis	[132]
			GDF11	Promotes apoptosis via the TGF pathway	[133]
	AD	Neuro2a APPSwe/Δ9 cells; AD patients ↑	PTGS2, CDK5	Represses neurite outgrowth but promotes cell apoptosis	[134]
MiR-103	IS	tMCAO rats ↑	VEGF	Regulates the angiogenesis	[135]
			NCX1	Regulates Ca^2+^ and Na^+^ homeostasis	[136]
			AXIN2	Reduces apoptosis through regulating the mitochondria-associated apoptosis signaling pathway	[137]
	AD	Mouse model of AD, CSF of AD patients ↓	PTGS2	Promotes neurite outgrowth and suppresses cells apoptosis	[138]
MiR-142	IS	AIS patients, primary mouse astrocytes and A172 cells ↓	TIPARP	Inhibits astrocyte activation via macroautophagy/autophagy	[139]
	AD	Aβ42-treated SH-SY5Y cells and AD patients ↓	PTPN1	Regulates VEGFR2 and Akt signaling	[140]
MiR-211	IS	OGD/R triggered C12 cells, penumbra of tMCAO mice ↓	PUMA	Promotes apoptosis	[141]
	AD	APPswe/PS1ΔE9 mice ↑	SIRT1/Nrf2/HO-1	SIRT1 pathway attenuates ROS-induced oxidative stress	[142]
			NUAK1	Regulates cortical neuron differentiation and survival	[143]
MiR-23-3p	IS	Cortex of C57/BL6 tMCAO mice ↑	CXCL12	Suppresses apoptosis	[144]
	AD	Cortex of APP/PS1 mice ↓	GSK-3β	Implicated in tau pathology	[145]
MiR-138	IS	OGD/R induced astrocytes ↓	p65	Essential for NF-κB dimerization	[146,147]
	AD	N2a/APP and HEK293/tau cell lines ↑	SIRT-1	Mediates synaptic function and APP processing via macroautophagy/autophagy	[148,149]
MiR-335	IS	tMCAO mice ↓	TIPARP	Astrocyte activation	[150]
			ROCK2	Promotes apoptosis	[151]
	AD	tissues of AD patients ↓	JNK3	Has key role in AD, mediated by many mechanisms	[152]
MiR-328	IS	Serum of IS patients ↓	No validated targets in IS conditions	Role in IS development is yet unknown, but connection between serum levels and short-term prognosis of stroke had been reported	[153]
	AD	SK-N-SH and SK-SY5Y cell lines ↓	BACE1	Required for the generation of all forms of Aβ, including Aβ42	[154]

* IS—ischemic stroke, AD—Alzheimer’s disease, tMCAO—transient middle cerebral artery occlusion, OGD/R—oxygen glucose deprivation/re-oxygenation, PBMCs—peripheral blood mononuclear cells, CSF—cerebrospinal fluid, Aβ—amyloid-β peptide, APP—amyloid precursor protein. Arrows (↑↓) indicate reported direction of expression change (↑—increase, ↓—decrease).

**Table 2:** CircRNAs associated with IS *.

circRNA	Differential Expression Data	Validated Targets	Reported Axis Function	References
ciRS-7 (CDR1as)	tMCAO mice ↓	miR-7/α-Syn (SNCA)	CDR1as overexpression suppressed α-Syn protein induction, promoted motor function recovery, decreased infarct size, curtailed the markers of apoptosis, autophagy and inflammation in the post-stroke brain	[178]
CircUCK2 (Circ_001128)	tMCAO mice ↓, OGD HT-22 cells ↓	miR-125b-5p/ GDF11	Upregulated circUCK2 levels decreased infarct volumes, attenuated neuronal injury and improved neurological deficits	[133]
CircHECTD1 (Circ_0000375)	tMCAO mice ↑, plasma of acute IS patients ↑, OGD-R A172 cells ↑	miR-142/ TIPARP	circHECTD1 leads to the inhibition of expression with subsequent inhibition of astrocyte activation via macroautophagy/autophagy	[139]
CircTLK1 (circ_0004442)	tMCAO mice ↓	miR-335/ TIPARP	Knockdown of circTLK1 decreased infarct volumes, attenuated neuronal injury, and improved neurological deficits	[150]
CircCDC14A	tMCAO mice ↓	miR-23a-3p/ CXCL12	Knockdown of circCDC14A suppressed MCAO-induced cerebral infarction and neurological damage, as well as the brain tissue damage and neuronal apoptosis in vivo	[144]

* IS—ischemic stroke, tMCAO—transient middle cerebral artery occlusion, OGD/R—oxygen glucose deprivation/re-oxygenation, arrows (↑↓) indicate reported direction of expression change (↑—increase, ↓—decrease).

**Table 3:** CircRNAs associated with AD *.

circRNA	Differential Expression Data	Validated Targets	Reported Axis Function	References
ciRS-7 (CDR1as)	Hippocampal region of AD patients ↓	miR-7/ UBE2A	Drives amyloid accumulation and the formation of senile plaque deposits	[131,170,182]
Circ_0000950 (circAKT1S1)	NGF-stimulated PC 12 cells, primary cerebral cortex neurons from rat embryo cells—no expression changes	miR-103/ PTGS2	Regulates neuron apoptosis, neurite outgrowth and IL-1β, IL-6 and TNF-α levels	[138]
CircHDAC9	Serum of AD patients and individuals with mild cognitive impairment ↓	miR-138/ Sirtuin-1	Mediates synaptic function and APP processing in AD	[148]
CircHIST1H4E (circ_0001588)	Streptozotocin-induced rat model of AD ↓	miR-211-5p/ SIRT1/Nrf2/HO-1	Control of reactive oxygen species production and malonaldehyde levels, regulates superoxide dismutase and glutathione levels	[142]
CircAXL (Circ_0002945)	AD serum and amyloid beta Aβ25-35-stimulated SK-N-SH cells and human primary neurons (HPNs) ↑	miR-328/ BACE1	Regulates apoptosis, neurite outgrowth and inflammatory cytokines in cellular AD models	[154]
		miR-431-5p/ TNFAIP1	Controls Aβ25-35-induced cell apoptosis and endoplasmic reticulum stress.	[183]

* AD—Alzheimer’s disease, Aβ—amyloid-β peptide, APP—amyloid precursor protein. Arrows (↑↓) indicate reported direction of expression change (↑—increase, ↓—decrease).
